# Preventive effects of a nutraceutical mixture of berberine, citrus and apple extracts on metabolic disturbances in Zucker fatty rats

**DOI:** 10.1371/journal.pone.0306783

**Published:** 2024-07-26

**Authors:** Mohamed Siliman Misha, Sandrine Destrumelle, Dylan Le Jan, Nahla M. Mansour, Lionel Fizanne, Khadija Ouguerram, Jean-Claude Desfontis, Mohamed-Yassine Mallem

**Affiliations:** 1 Nutrition, Pathophysiology and Pharmacology (NP3) Unit, Oniris, Nantes Atlantic College of Veterinary Medicine, Food Science and Engineering, Nantes, France; 2 Department of Chemistry of Natural and Microbial Products, Division of Pharmaceutical Industries, National Research Centre, Giza, Egypt; 3 Laboratoire HIFIH UPRES EA 3859, SFR ICAT 4208, Université d’Angers, Angers, France; 4 INRAE, UMR 1280, Physiopathology of Nutritional Adaptations, Nantes, France; Arizona State University, UNITED STATES OF AMERICA

## Abstract

**Background:**

The prevention of obesity represents a major health and socio-economic challenge. Nutraceuticals are regularly highlighted for their beneficial effects in preventing the metabolic disturbances associated with obesity. However, few studies have described the combined action of nutraceutical mixtures combining polyphenols with alkaloids.

**Objective:**

The aim of this study was to evaluate the effects of long-term dietary supplementation with a mixture of Berberine, Citrus and Apple extracts (BCA) in the primary prevention of obesity and its metabolic and vascular complications in the obese Zucker rat, a spontaneous model of genetic obesity and insulin resistance.

**Methods:**

Sixteen 8-week-old obese Zucker male rats were randomly divided into two groups: all rats received oral gavage daily either with water, untreated obese (U-ObZ) or BCA (BCA-ObZ) mixture for thirteen weeks. Morphological and metabolic parameters were measured along the study. Cumulative concentration-response curves to insulin, acetylcholine and phenylephrine were determined on isolated thoracic aorta. Colon permeability measurements were performed using the Ussing chamber technique. Fecal samples collected at the beginning and the end of the protocol were used as a template for amplification of the V3-V4 region of the 16S rDNA genes.

**Results:**

BCA supplementation reduced weight gain (p<0.05) and food intake (p<0.05) in the BCA-ObZ group rats compared to the U-ObZ group rats. It also improved glucose tolerance (p<0.001) and decreased fasting insulin and Homeostasis model assessment index (p<0.05). Through *ex vivo* experiments, the BCA mixture enhanced significantly aortic insulin relaxation (p<0.01), reduced α_1_-adrenoceptor-mediated vasoconstriction (p<0.01), and decreased distal colon permeability. Moreover, short-chain fatty acid producers such as *Bacteroides*, *Blautia*, and *Akkermansia* were found to be increased by the BCA mixture supplementation.

**Conclusion:**

The results showed that a 13-week-supplementation with BCA mixture prevented weight gain and improved glucose metabolism in obese Zucker rats. We also demonstrated that BCA supplementation improved vascular function, colonic barrier permeability and gut microbiota profile.

## 1. Introduction

Obesity is a multifactorial disease. These include genetics, psychological factors (stress, depression), medical factors (the use of certain medications such as beta-blockers, corticoids and antidepressants), as well as lifestyle factors including a sedentary lifestyle and excessive diet [1–3]. Particularly ultra-processed products, resulting in excessive and dysfunctional accumulation of body fat [2,4,5]. Its prevalence is estimated to reach 13% (2023) and 15.9% (2022) worldwide and in the European population respectively [3,6,7]. The pathological feature of obesity is mainly an increase of visceral fat associated with an increased risk of developing metabolic syndrome [7–9], including the obesity-linked meta-inflammation [10], being an important contributor to insulin resistance and alteration of carbohydrate and lipid homeostasis [10–12]. Assuming the multifactorial nature of obesity, the complexity and multiplicity of mechanisms and mediators involved (*i*.*e*. oxidative stress, inflammation, arterial hypertension) [12–14], public health organizations and scientific journals agree that multi-component and/or multi-disciplinary interventions are the best option for treating obesity [15–17]. Therefore, therapeutic strategies focused on multi-ingredient-based interventions, including mixtures of nutraceuticals, can be considered more appropriate, due to the cumulative efficacy targeting multiple pathways simultaneously (targeting multiple pathways simultaneously) of such products to overcome the obesity [18].

Nutraceuticals are foods or food-derived products that have health benefits on the main components of the metabolic syndrome [11,18,19]; they have been shown to inhibit lipogenesis and promote lipolysis in the adipose tissue [20–22], to improve insulin sensitivity [18,23,24] and reduce appetite [25,26]. In this study, a nutraceutical blend of Berberine and hydro-alcoholic extracts of Citrus and Apple (BCA) was selected to explore its activity against obesity and obesity-related disorders. The individual pharmacological activities of these ingredients have been previously described. Berberine, an isoquinoline plant alkaloid [19,27] has been paid more attention due to its anti-obesity activities like insulin sensitizing [28,29] and anti-inflammatory [30–32] effects, and its ability to increase energy expenditure by lipolytic and thermogenic actions [21]. Citrus flavonoids have been reported to exert anti-obesity effects due to reduction of appetite, regulation of metabolism and fat storage by mechanisms involving in part a role of hesperidin [21,33,34]. They also have shown anti-inflammatory and antioxidant properties [20,33]. Previously studies have also proven that apple extracts exhibit a favorable effect on metabolic health thanks to the various components of the skin and apple pulp (pectins, phenolic compounds, flavonoids: quercetin, phlorizin [22,35,36]. The metabolic protection of (via the effects of nutraceuticals) comprises the activation of satietogen mechanisms, the limitation of the gut absorption of carbohydrates and lipids and the decrease of ectopic fat accumulation [34–36].

This study investigated the possible synergistic effects of BCA in terms of primary prevention of obesity and its metabolic and vascular complications in the obese Zucker rat, a spontaneous model of genetic obesity and insulin resistance [37,38]. We hypothesize that long-term supplementation with BCA mixture can prevent obesity and related metabolic and vascular issues through the combined effects of its main ingredients, implying on the one hand, an action on effectors acting directly on weight loss, and on the other hand, the implementation of anti-inflammatory and regulatory actions of metabolism.

## 2. Material & methods

### 2.1. Animals

Sixteen 8-week-old male obese Zucker (fa/fa) rats (weight range: 349.20±5.09 g) included in this study were purchased from Janvier Labs^®^ (Le Genest-Saint-Isle, France). Rats were acclimatized for one week before being randomly assigned to 2 groups: Untreated Obese Zucker (U-ObZ) receiving vehicle (water) and the BCA-Treated Obese Zucker (BCA-ObZ) group receiving the orally administered Berberine and hydro-alcoholic extracts of Citrus and Apple (BCA) mixture (200 mg/kg/day, the dose comprises 100 mg berberine, 50 mg citrus extract and 50 mg apple extract.) for 13 weeks. The environment of their housing room was controlled according to the requirements of the European standard ETS 123, relating to the housing of animals in experimental animal facilities. Thus, the light is regulated with a 12-hour day/night cycle, the temperature is 22 ±2°C, the hygrometry is around 50%, ventilation and noise pollution are reduced to a minimum. Standard rodent food (Serlab^®^, Montataire, France) and drinking water were provided *ad libitum*. Ethical approval of the project was obtained beforehand and all experiments were performed in accordance with the institutional guidelines of the Ethics Committee of Pays de la Loire, France (ministerial authorization, APAFIS#27136–2020090716464179 v2).

### 2.2. Apple and citrus extracts and drugs

Apple extract (Appl’In^®^) was provided by Diana Naturals (Antrain, France). This is a fine powder of a polyphenolic extract obtained by hydro-ethanolic extraction method from apple (*Malus domestica Borkh*) standardised for its content in phloridzin (at least 5%). The specific batch (SD01290004) used in the study contained 5.33% of phlorizin, with a total polyphenols content (hydroxycinnamic acids, flavonols, flavanols) of 87.6% of the final product expressed as catechin equivalents.

Citrus extract (*Citrus aurantium L)* was supplied by Naturex (Caronno Pertusella, VA, Italy). The extract is a granulated powder obtained by water extraction from *Citrus aurantium* pericarps (100 mg of extract are equivelent to an average of 290 mg of dry citrus aurantium pericarps). The specific batch used in the study (EC834725) contained around 35% bioflavonoids, including rutin, hesperidin, quercetin and amentoflavone, with hesperidin as the main component (around 37%).

Berberine chloride (100 mM stock solutions of berberine chloride in warm distilled water [39])was obtained Sigma Aldrich Chimie^®^ (Saint Quentin-Fallavier, France).

Sodium pentobarbital solution was purchased from CEVA Santé Animale (Libourne,France); phenylephrine hydrochloride, acetylcholine chloride, insulin (prepared in 0.01 M hydrochloric acid solution at a concentration of 20 mg/mL) and berberine chloride were supplied by Sigma Aldrich Chimie^®^ (Saint Quentin-Fallavier, France).

All drugs were prepared as stock solutions in distilled water, with the exception of berberine chloride wich was dissolved in warm distilled water [39].

### 2.3. Physiological parameters

Body weight, abdominal circumference, total length and food and water consumption of all rats were monitored weekly. The BMI was calculated according to the following formula: BMI = body weight (g)/length^2^ (cm^2^). The adiposity index was calculated as (total body fat/final body weight) × 100.

Before and at the end of the protocol, blood collection was performed after a 4-h physiological fasting. Plasma sampling were stored at -80°C for subsequent determination of plasma levels of total cholesterol (TC), triglycerides (TG) and high-density lipoprotein cholesterol (HDL-C) by Nantes Hospital Center. Moreover, plasma levels of insulin were quantified by Nantes Laboniris laboratory.

### 2.4 Glucose tolerance test and biochemical measurements

An oral glucose tolerance test was performed (2 g/kg body weight, *per os*) in 4-hour fasted rats at baseline, 6, 9 and 13 weeks. Blood was collected from the tail vein at T0, 15, 30, 45, 60, 90 and 120 minutes and then glucose concentration was determined with a calibrated rodent blood glucose meter (StatStrip, Nova Biomedical^®^). The area under the curve (AUC) was calculated using GraphPad PRISM^®^ software (version 9.3.0) using the trapezoidal method.

Homeostasis Model Assessment of Insulin Resistance (HOMA-IR) was calculated using the following equation: [(fasting blood glucose (mmol/l) x fasting plasma insulin (μU/ml)]/22.5] at the beginning and end of the protocol [40].

### 2.5. Vascular reactivity experiments

After the sacrifice (pentobarbital overdose) of the rat, the thoracic aorta was removed, cleaned of fat and connective tissue, and placed in cold Krebs solution. The artery was cut into 3-4-mm rings that were mounted on triangular wire supports and suspended in 10-mL organ baths containing Krebs solution, maintained at 37°C and gassed with carbogen (95% O_2_ and 5% CO_2_) [21]. The isometric tension was measured continuously with an automated isometric transducer system (EMKAbath4, EMKA technologies, France) and recorded using data acquisition software (iox version 2.9.10.25. EMKA technologies, France).

As previously described [41], the aorta rings were stretched at a resting tension of 2 g in 0.5 g steps and allowed to equilibrate for 15 min per step. Then, Cumulative Concentration-Response Curves (CCRCs) to phenylephrine (Phe), an α_1_-adrenoceptor agonist (10^−9^ to 3.10^−5^ M), acetylcholine (Ach), a muscarinic receptor agonist (10^−9^ to 3.10^−5^ M), and insulin (Ins) (10^−9^ to 10^−5^ M) were performed. Ach and Ins CCRCs were constructed in Phe-precontracted rings. Contractile responses to Phe were measured as the increase in tension expressed as a percentage of the peak rise in tension caused by 80 mM KCl. The relaxant responses to Ach and Ins were calculated as the percentage change in the maximal tension of vessel rings after addition of phenylephrine.

### 2.6. Colonic reactivity experiments

After the sacrifice, the colon is removed and placed in cold Krebs solution previously carbogenized (95% O_2_, 5% CO_2_). After being dissected, fat-free and its contents rinsed with cold Krebs, the colon was cut into 0.5 to 1 cm longitudinal segments (2 proximal segments approximately 3 cm from the cecum and 2 distal segments approximately 2 cm from the rectum), segments were mounted in 10-mL organ baths containing Krebs solution, maintained at 37°C and gassed with carbogen. The isometric tension was measured continuously with an automated isometric transducer system (EMKAbath4, EMKA technologies, France) and recorded in a data acquisition software (iox version 2.9.10.25. EMKA technologies, France).

Once the system is set up, a stabilization period (60 minutes) is necessary before performing the non-cumulative concentration-response curves (CCRCs). During this time the segments are stretched at a resting tension of 1 g, including a systematic flush every 15 minutes and a return to basal tension. The segment was evaluated stable after obtaining two reproducible responses to KCl (80 mM).

Then, to evaluate the colonic reactivity, non-cumulative curves are performed with KCl and Carbachol with or without prior incubation with short-chain fatty acids (SCFA): Butyrate (5mM) and propionate (5mM).

### 2.7. Colonic paracellular permeability

Following the same sampling and dissection protocol as for colonic reactivity, the segments were opened in half and placed on sliders (Area = 0.031 cm^2^) and finally mounted in Ussing chambers (Physiologic Instruments, San Diego, CA, USA) in 3 mL of Krebs maintained at 37°C with carbogen (95% O_2_ and 5% CO_2_). After 30 min of equilibration, a volume of Krebs (100 μL), from the mucosal side was collected and then replaced with 100 μL FITC -Sulfonic Acid (FSA, 400 Da). In order to evaluate the paracellular permeability, a volume of Krebs (150 μL) was taken from the serous side and replaced with fresh Krebs every half hour for 3 hours. Permeability was determined by the fluorescence slope related to time.

### 2.8. Gut microbiota analysis

#### Library preparation and sequencing

The polymerase chain reaction (PCR) technique was used to amplify regions V3-V4 of the gene coding for 16S ribosomal RNA using primers 341F and 785R [42]. Magnetic AMPure XP beads (Beckman Coulter, France) were used to clean the amplicons before adding double indexes and sequencing adapters using Illumina’s Nextera XT index kit (Illumina, USA). Each library was cleaned and quantified by fluorimetry (Qubit^®^ 2.0 Fluorometer), normalized and pooled. The pooled library was denatured prior to sequencing (2x250 pair-end, chemistry v2) using an Illumina MiSeq (Illumina, USA).

#### Data processing

A bioinformatics pipeline developed by Biofortis and based on Dadaist2 software [43] was used for sequence analysis. Basically, after demultiplexing indexed reads, single-read sequences were matched for each sample into longer fragments and cleaned up. Amplicon variant sequences (AVSs) were obtained after quality filtering and modeling of sequencing errors. In order to determine bacterial community profiles, a taxonomic assignment of these AVSs was carried out.

### 2.9 Histological analyses

Two liver lobes were sliced and fixed in 4% paraformaldehyde for 24 hours, then embedded in paraffin. Staining of the 5 μm-thick sections was then performed with hematoxylin-eosin-saffron (HES) solution or 0.1% picrosirius red (steatosis and fibrosis zone). These samples were then analyzed with an automatic threshold technique using an algorithm developed in the HIFIH laboratory (EA 3859, Angers, France) as previously described [44].

### 2.10. Statistical analysis

Results were expressed as the mean ± standard error of the mean (SEM) of “n” experiments with “n” corresponding to the number of rats studied. The influence of BCA treatment on morphometric, metabolic and microbiota parameters was evaluated with Two-Way ANOVA, unpaired t test or Mann-Whitney test.

Ach and Phe CCRCs are drawn using GraphPad PRISM^®^ software (version 9.3) and analyzed by a nonlinear mixed-effects model (NLME) using R software (RStudio 2022.12.0+353).

Data from insulin CCRCs are analyzed by a linear mixed-effects model (LME) using R software [45].

For each statistical test, a value of P<0.05 was considered to be significant.

## 3. Results

### 3.1 Effect of BCA treatment on body weight and metabolic parameters

**Fig 1:**
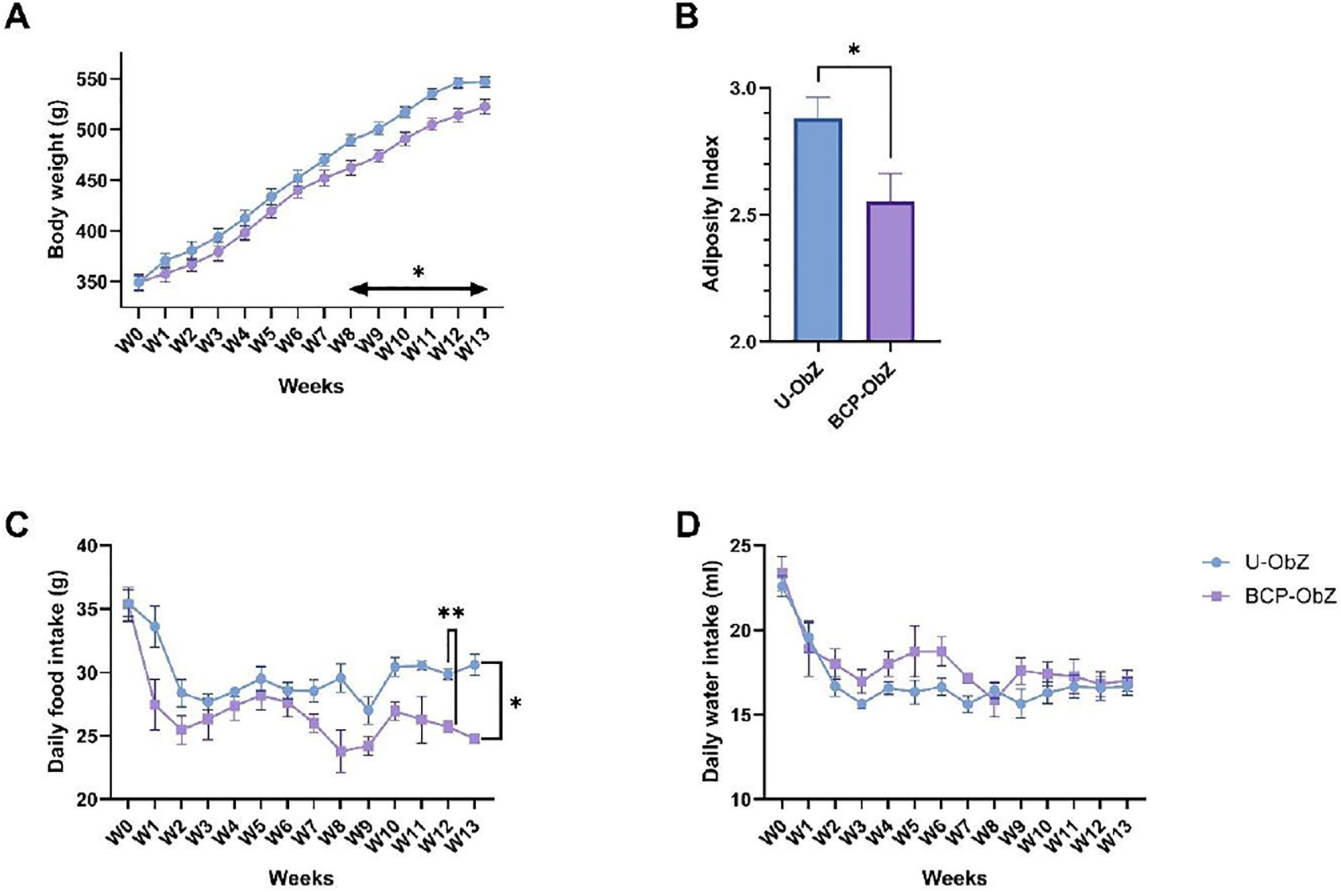
Effect of BCA mix on body weight (A), adiposity index (B), food intake (C) and water intake (D). Data were expressed as mean ± SEM. A Mann-Whitney test or Two-way ANOVA test were used for statistical analysis. n = 8 for each group. * p<0.05; ** p<0.01 BCA-ObZ vs. U-ObZ. U-ObZ: Untreated Obese Zucker; BCA-ObZ: BCA-Treated Obese Zucker.

The administration of the BCA mixture significantly reduced weight gain after 8 weeks of treatment compared to U-ObZ rats (p<0.05) (Fig 1A). Furthermore, after 13 weeks of supplementation, the weight gain reduction was accompanied by significant decreases in abdominal circumference (p<0.05), in BMI (Body Mass Index) (p<0.05, Table 1), and in adiposity index (p<0.05) in BCA-ObZ rats (Fig 1B). These reductions were also associated with a decrease in food intake (p<0.05) while the water consumption was not affected (Fig 1C & 1D).

**Table 1:** Effect of BCA supplementation on weight, abdominal circumference, fasting glycaemia, insulin, plasma lipids and hepatic transaminases.

	U-ObZ		BCA-ObZ		p-value (between groups)	
	Baseline	13^th^ week	Baseline	13^th^ week	Baseline	13^th^ week
Weight (g)	349.20 ± 6.50	545.90 ± 5.70	349.30 ± 8.27	516.70 ± 8.15	ns	*0.0281
Abdominal circumference (cm)	15.41 ± 0.20	20.45 ± 0.18	15.33 ± 0.17	19.70 ± 0.27	ns	*0.034
BMI	0.7539 ± 0.01	0.86 ± 0.01	0.75 ± 0.01	0.82 ± 0.01	ns	*0.0499
Fasting glycaemia (mg/dL)	95.38 ± 2.22	109.6 ± 2.88	94.25 ± 3.519	103.0 ± 3.09	ns	ns
Fasting insulinemia (ng/mL)	4.806 ± 0.58	9.301 ± 1.21	4.93 ± 0.54	6.252 ± 0.73	ns	*0.0379
Triglycerides (g/L)	2.563 ± 0.19	3.961 ± 0.38	1.80 ± 0.19	4.65 ± 0.38	**0.0064	ns
Total cholesterol (g/L)	3.63 ± 0.08	5.874 ± 0.27	3.5 ±0.15	5.194 ± 0.26	ns	ns
HDL-C (g/L)	2.885 ± 0.05	3.923 ± 0.19	2.838 ± 0.12	3.59 ± 0.074	ns	ns
ASAT (IU/L)	89.93 ± 4.15	89.53 ± 10.67	99.23 ± 7.02	107.7 ± 15.87	ns	ns
ALAT (IU/L)	140.8 ± 7.18	94.67 ± 13.24	154.4 ± 10.37	118.8 ± 28.46	ns	ns
ASAT/ALAT	0.65 ± 0.02	0.96 ± 0.04	0.65 ± 0.04	1.11 ± 0.19	ns	ns

ns: Not significant;

* p<0.05;

** p<0.01 BCA-ObZ vs. U-ObZ.

U-ObZ: U-ObZ: Untreated Obese Zucker; BCA-ObZ: BCA-Treated Obese Zucker; BMI: Body Mass Index; HDL-C = high-density lipoprotein, ASAT = aspartate aminotransferase; ALAT = alanine aminotransferase.

We found an increase in total cholesterol (TC) and high-density lipoprotein cholesterol (HDL-C) in both groups between baseline and week 13 in the U-ObZ rats (p<0.05) and BCA-ObZ rats (p<0.05) groups. Nevertheless, no significant differences emerged between the groups at week 13 (Table 1).

### 3.2. Effect of BCA treatment on glucose and insulin levels

**Fig 2:**
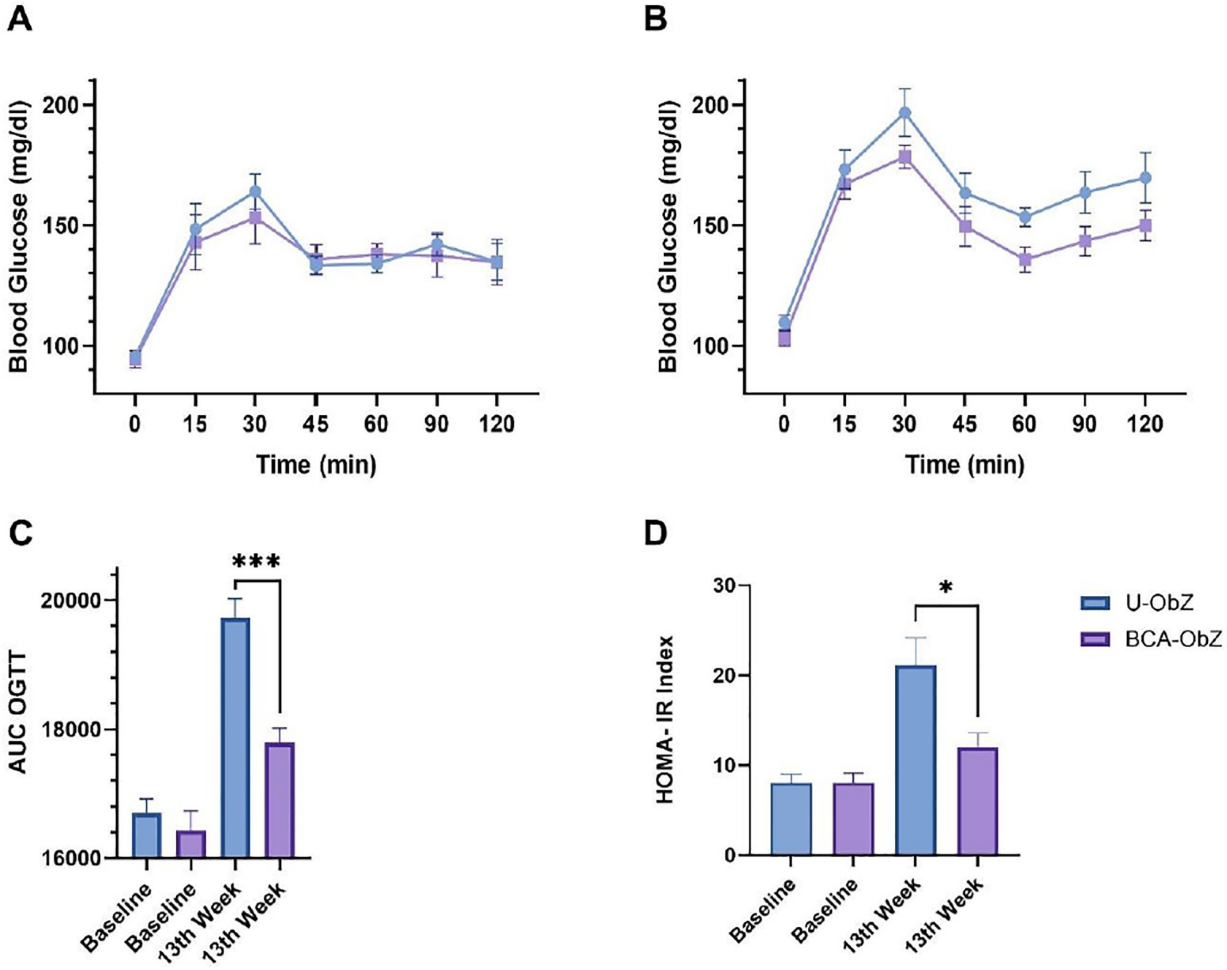
Effect of BCA blend on glucose and insulin metabolism. Glucose metabolism was assessed by oral glucose tolerance test (OGTT), performed at the beginning and end of the protocol. After a glucose bolus, plasma glucose was measured at baseline (A) and at week 13 of the protocol (B). The area under the curve (AUC) of OGTT was calculated at baseline and at week 13of the protocol (C). Insulin metabolism was assessed by calculating the insulin resistance assessment model (HOMA-IR) (D). Data were expressed as mean ± SEM. A Mann-Whitney test or Unpaired t test were used for statistical analysis. n = 8 * p<0.05; *** p<0.001 BCA-ObZ vs. U-ObZ. U-ObZ: Untreated Obese Zucker; BCA-ObZ: BCA-Treated Obese Zucker.

The basal levels (baseline) showed no significant difference between groups in OGTT AUC and blood glucose levels over a 2-hour period after testing (Fig 2A & 2B). In addition, no difference was observed in fasting blood glucose levels. After 13 weeks, fasting blood glucose levels, AUC, and 2-hour post-OGTT blood glucose levels increased for both groups. However, the BCA mixture group had significantly lowered AUC and HOMA-IR index compared to the control group (Fig 2C & 2D).

### 3.3 Effect of BCA treatment on hepatic metabolic parameters

There was no significant change in ASAT levels from baseline to the end in either the untreated or BCA-treated group. However, the ALAT level decreased significantly in the untreated group (p = 0.0331), and decreased non-significantly in the BCA-treated group compared to baseline values. There was no significant difference observed between the two groups for the ASAT/ALAT ratio (Table 1).

Furthermore, there was no significant difference observed between the untreated and BCA-treated groups for the weighted fibrosis area (S1A Fig). While there was a trend towards a decrease in the steatosis area in the BCA-ObZ group, this difference was not significant (S1B Fig).

### 3.4. Vascular reactivity

**Fig 3:**
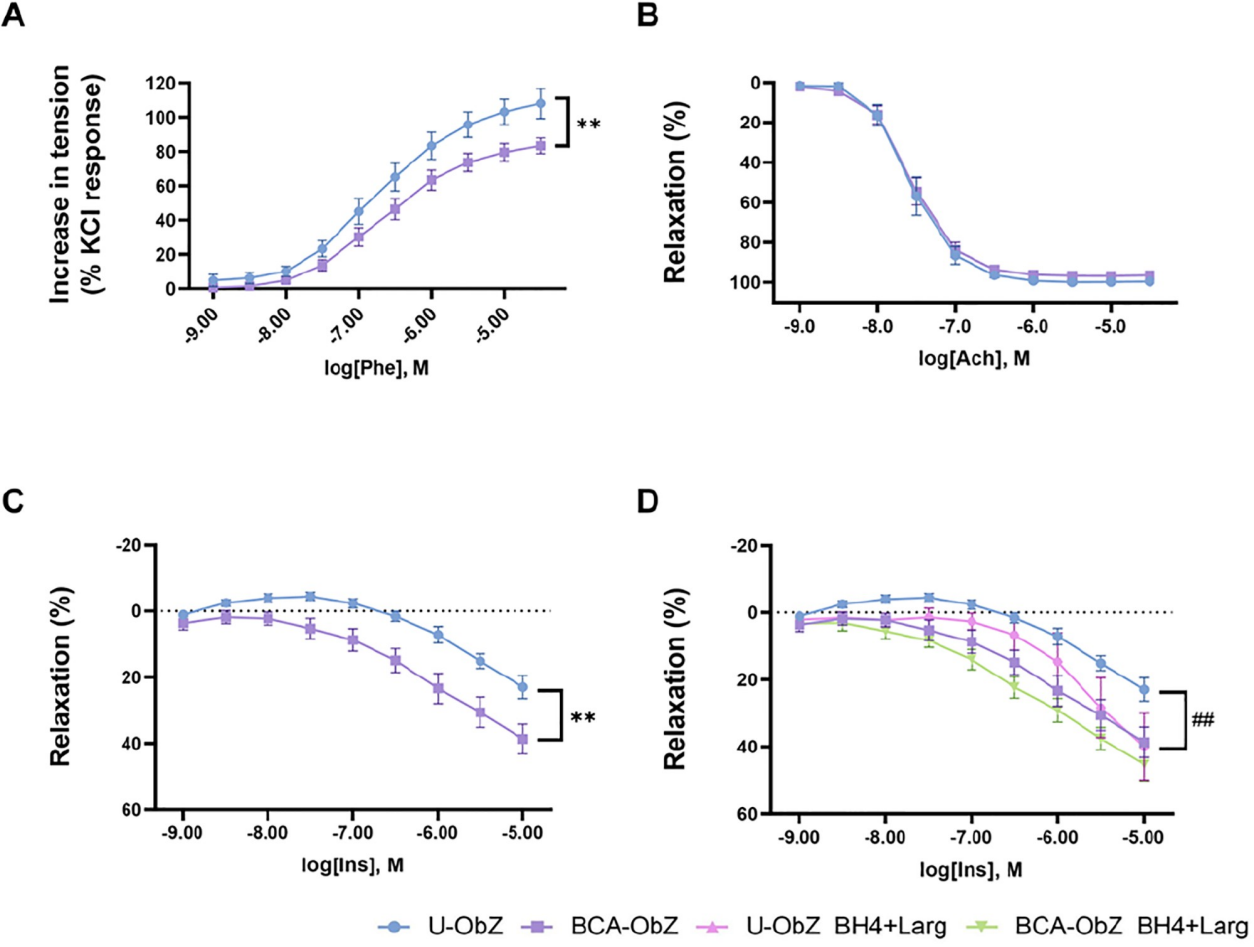
Effect of BCA treatment on aortic vasoreactivity. CCRCs were constructed to assess aortic contractile responses using phenylephrine (Phe, 10^−9^ to 3.10^−5^ M (A) and relaxation responses using acetylcholine (Ach, 10^−9^ to 3.10^−5^ M) (B), insulin (Ins, 10^−9^ to 10^−5^ M) (C) and insulin (Ins, 10^−9^ to 10^−5^ M) with or not incubated BH4 (10^−5^ M) and L-Arg (10^−3^ M) (D). Percentages of contraction and relaxation calculated are relative to maximal changes from pre-contraction produced by KCl and Phe, respectively. Data are expressed as mean ± SEM and determined using NLME for Phe and Ach and LME for Ins. n = 6–8 each group. **p<0.01 BCA-ObZ vs. U-ObZ. ## p<0.01 U-ObZ+BH4+Larg vs. U-ObZ. U-ObZ: Untreated Obese Zucker; BCA-ObZ: BCA-Treated Obese Zucker; BH4: Tetrahydrobiopterin; L-Arg: L-arginine; NLME Non-linear Mixed Effect; LME: Linear Mixed effect.

The maximal contractile response to Phe in aortic rings from BCA-treated group was significantly lower (Emax = 78.23±7.49) than that in the U-ObZ group (Emax = 105.8±8.5) (p<0.01) (Fig 3A). In the present study, we evaluated the effects of long-term treatment with BCA on endothelium-dependent aortic relaxation using Ach and Ins. Our results showed that Ach-induced relaxation was similar in aortic rings from control (Emax = 99.2±0.8; pD2 = 7.6±0.1) and BCA group (Emax = 99.2±0.8; pD2 = 7.6±0.1) (Fig 3B). However, we found that the maximal relaxant response to Ins in aortic rings from the BCA-ObZ group (maximal response to the higher concentration was 38.6±9.5) was significantly higher than that in the control group (maximal response to the higher concentration was 22.9±6.2) (p<0.01) (Fig 3C). The treatment with the eNOS cofactors BH4 and L-Arg also enhanced the response to Ins in both untreated (maximal response to the higher concentration was 39.9±11.5; p<0.05) and BCA-treated groups (maximal response to the higher concentration was 45.2±10.8) (Fig 3D).

### 3.5. Colonic reactivity

**Fig 4:**
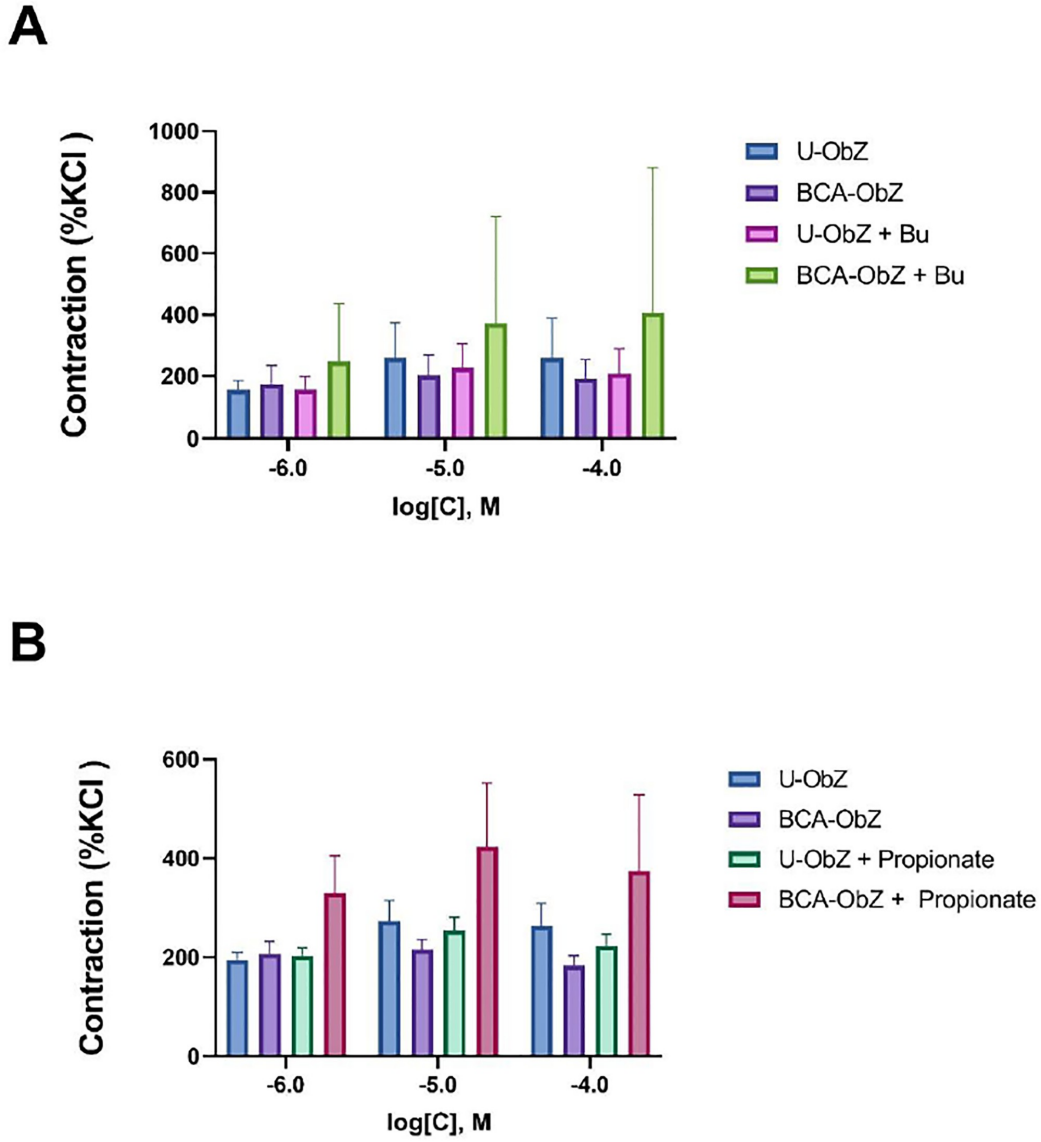
Effect of BCA mixture on carbachol-induced contraction in isolated segments of distal colon from control and BCA-treated groups. The responses were obtained after pretreatment with sodium butyrate (A) or propionate (B). Data are expressed as mean ± SEM. LME was used for statistical analysis. n = 8 for each group. U-ObZ: Untreated Obese Zucker; BCA-ObZ: BCA-Treated Obese Zucker; LME: Linear Mixed effect.

In the U-ObZ group, Carbachol (1 mM-100 mM) induced a contractile response of the isolated distal colonic segments that was slightly, although not significantly decreased at the higher concentrations (10 mM and 100 mM) by BCA treatment (Fig 4A). Distal colonic segments seem to exhibit a higher contractile response to Carbachol after treatment with Butyrate only in the BCA-ObZ group (Fig 4A). A similar pattern of Carbachol-induced response was seen in the BCA-ObZ group when the distal colonic segments were pretreated with propionate (Fig 4B).

### 3.6. Colonic paracellular permeability

**Fig 5:**
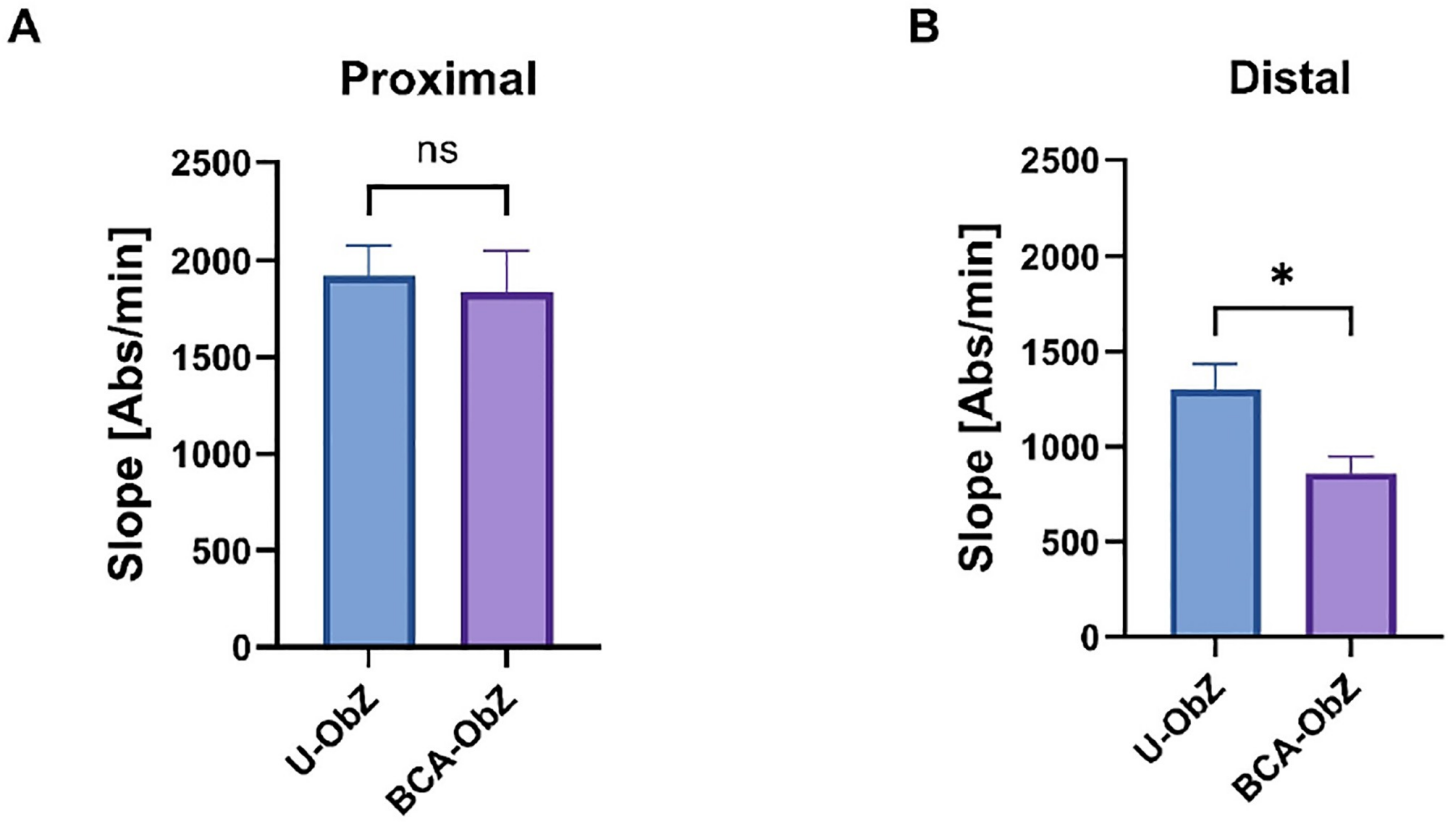
Permeability to fluorescein sulfonic acid was measured using Ussing chambers in the proximal colon (A) and in the distal colon (B). Data are expressed as mean ± SEM. The Mann-Whitney test was used for statistical analysis. n = 8 for each group. ns: Not significant; * p<0.05 BCA-ObZ vs. U-ObZ. U-ObZ: Untreated Obese Zucker; BCA-ObZ: BCA-Treated Obese Zucker.

The paracellular permeability of the proximal colon segments was studied in the Ussing chamber, with results indicating no significant difference in permeability when assessed over 3 hours (Fig 5A). However, in the distal colon segment, the addition of the BCA mixture resulted in a 51% reduction in FD4 flow compared with the U-ObZ group (Fig 5B).

### 3.7. Cecum morphometric parameter

We compared the morphometry of the cecum and the *ratio* of cecal weight to body weight between the BCA-ObZ and U-ObZ groups. Regarding morphological differences, our observations revealed a significant disparity, as depicted in S2A Fig. Furthermore, the cecal weights were notably greater in the BCA-ObZ group compared to the U-ObZ group (p<0.001; S2B Fig).

### 3.8 Microbiome analysis

A total of thirty-two fecal samples were collected from the two rat groups. Fecal samples were collected at the beginning and at the end of the experiment to find out the effect of the treatment using the BCA mixture on the microbiota composition. Thus, the DNA was extracted from the fecal samples from each rat at the start Week 0 and at the end week 13 and used in the amplification of V3-V4 variable region of 16S rRNA genes.

**Fig 6:**
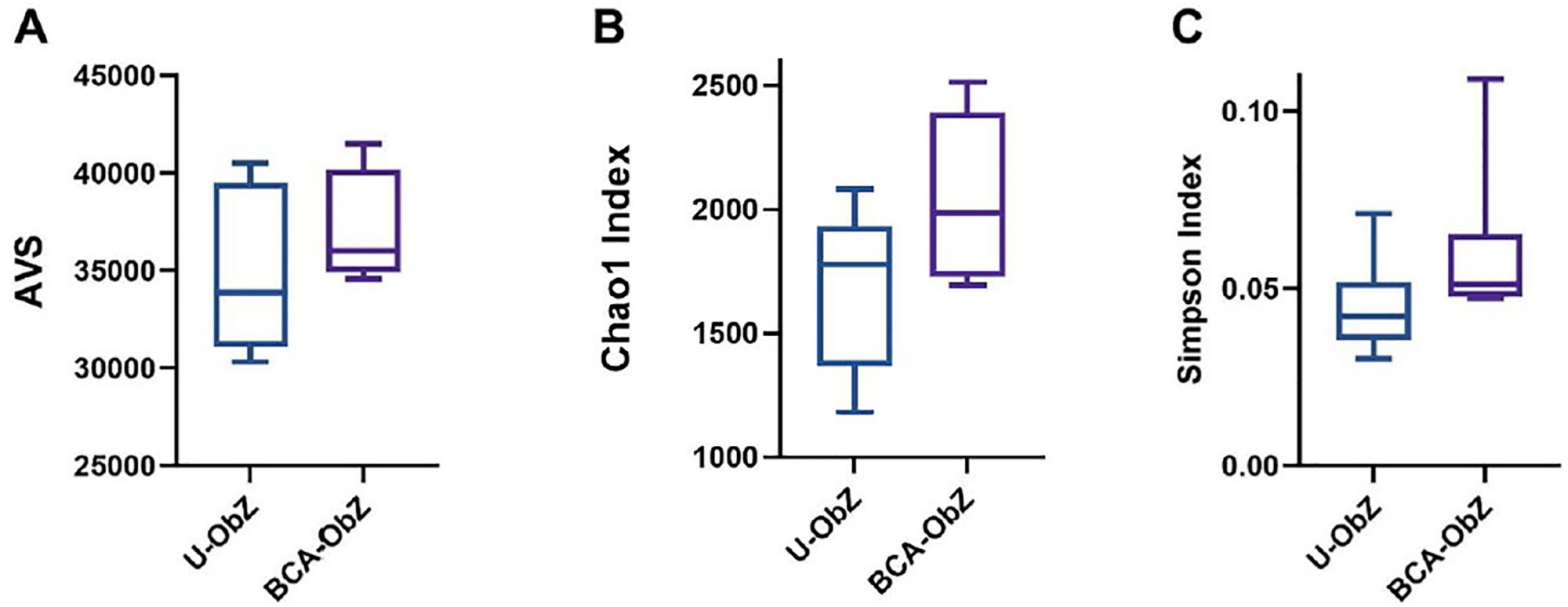
The panels represent the alpha diversity measures where; A) presents the observed library (total number of AVS observed); B) presents Chao1 index is the richness estimators (estimate the total number of AVS present in a community); C) presents Simpson is microbial indexes of diversity. Boxes span the first to third quartiles; the horizontal line inside the boxes represents the median. U-ObZ: Untreated Obese Zucker; BCA-ObZ: BCA-Treated Obese Zucker.

The raw sequence reads in fastq files were quality filtered and pre-processed before assessment and classification using specific software and database as mentioned in the materials and methods section. The results showed library size extended from 31,702 to 47,345 reads across the sequence samples, and this consider an adequate reads per sample for taxonomic analysis. Total number of AVS’s observed ranged from 123 to 414 which fit with richness estimation by Chao1 and ACE. The taxonomic levels include 11 phyla, 20 classes, 33 orders, 65 families, and 199 genera (Fig 6).

Alpha-diversity was implemented towards investigate the species complexity according to Fisher, Gini_Simpson, InvSimpson, PD, Pielou, Shannon and Simpson. The Shannon alpha-diversity index extended from 2.85 to 4.59; detected richness extended from 20.32 to 62.74 and Pielou’s evenness extended from 0.59 to 0.76 (Fig 6).

**Fig 7:**
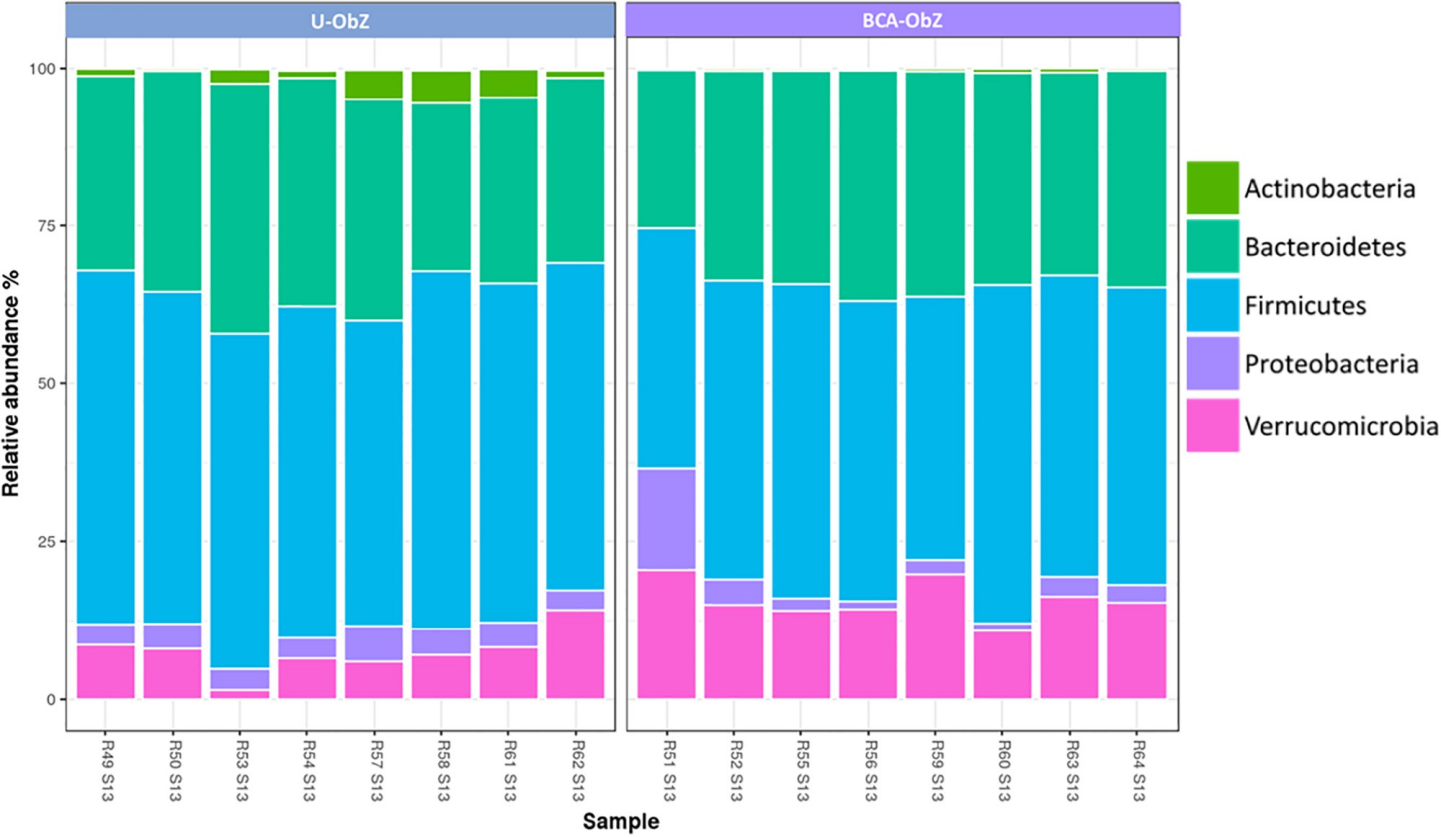
The phylum composition of microbiome after the 13^th^ week of experimentation. U-ObZ: Untreated Obese Zucker; BCA-ObZ: BCA-Treated Obese Zucker.

At phylum level, 11 phyla were initially identified. The most represented phyla were Bacteroidetes and Firmicutes, representing an equal average relative abundance of around 45% of all samples at the start of the experiment, followed by Akkermansiaceae representing 4%, Proteobacteria 3.5% and Actinobacteria 2.5% (Fig 7).

More specifically, at the start of the experiment (baseline the U-ObZ group showed an average relative abundance for the following five phyla: Bacteroidetes 45%, Firmicutes 44.81%, Akkermansiaceae 4.26%, Proteobacteria 3.77% and Actinobacteria 2.58%. At the end of the experiment (13 weeks), this group presented 31.09% Bacteroidetes, 54.11% Firmicutes, 7.99% Akkermansiaceae, 4.77% Proteobacteria and 2.61% Actinobacteria. Similarly, at the start of the experiment, the BCA-Obz group showed 45.81% Bacteroidetes, 42.95% Firmicutes, 5.69% Akkermansiaceae, 3.94% Proteobacteria and 1.60% Actinobacteria (S3 Fig). At the end of the experiment, this BCA-ObZ group contained 30.71% Bacteroidetes, 47.82% Firmicutes, 16.72% Akkermansiaceae, 4.30% Proteobacteria and 0.43% Actinobacteria (S3 Fig). As the results show, at the end of the experiment, the groups treated an increase in Akkermansiaceae (16.72%), followed by Firmicutes, while Actinobacteria decreased by only 0.43%.

Bacteria identified by microbiome analysis are shown in Krona plots, representing relative abundance of microbial species detected in BCA- ObZ week 0 and week 13 (S3 Fig).

At 0 week (baseline), the fecal microbiome consists of *Prevotellaceae* 28% (*Prevotella copri* 89%), *Clostridia* 28% (Romboutsia timonensis 14%), *Lactobacillales* 7% *(Lactobacillus johnsonii* 48%), *Akkermansiaceae* 6% (*Akkermansia muciniphila*), *Acidaminococcaceae* 4% (*Phascolarctobacterium faecium*), *Erysipelotrichales* 4% *(Turicibacter sanguinis* 92%), *Sutterellaceae* 3% (*Parasutterella excrementihominis*), *Ruminococcaceae* 3%.

The composition of microbiome showed changes after the 13 weeks as follows; *Prevotellaceae* 23%, ***Erysipelotrichaceae* 14%** (*Turicibacter sanguinis*), *Lachnospiraceae* 12%, *Lactobacillales* **10%** (*Ligilactobacillus apodemi* 42%, *Limosilactobacillus reuteri* 25%, ***Lactobacillus johnsonii* 18%**, *Lactobacillus gasseri* 15%) ***Akkermansia muciniphila* 8%**, ***Ruminococcaceae* 6%** (S3 Fig).

## 4. Discussion

In the present study we evaluated the potential effectiveness of a nutraceutical supplementation, comprising a combination of berberine and extracts of citrus and apple, in mitigating the metabolic and functional disorders associated with obesity. To this end, we used the Zucker rat as a genetic model of obesity and insulin resistance. The untreated obese (fa/fa) Zucker rats showed a significant increase in weight from week 0 to week 13 that is clearly linked to features of the metabolic syndrome (insulin resistance, dyslipidemia) as previously described [37,46,47]. Collectively all the studies cited above have explored the individual activities of berberine, apple extract or citrus extract, and have shown that these natural products exhibited anti-obesity properties that involved multi-targets and pathways (ie reducing fat mass, improving insulin sensitivity, and increasing thermogenesis and energy expenditure and lipid breakdown). However, to the best of our knowledge, little is known about the effects due to their combination and their long-term combined use on obesity and obesity-related metabolic and functional alterations. Whether the potential complementary actions on the lipid/glucide metabolism regulation, reduction of energy absorption or increase in its expenditure, or appetite suppression, attributed to berberine, citrus extract and apple extract respectively, may achieve a greater nutraceutical effect in obese Zucker rat is still unknown and remains to be determined.

In this study we aimed to evaluate whether polyphenols from apple and citrus extracts combined with the alkaloid berberine, when consumed together, could target synergistically the obesity and related disorders in obese Zucker rat. One of the main findings of the present study is that long-term supplementation with BCA mixture reduced weight gain, abdominal circumference and adiposity index in treated rats compared to the untreated obese Zucker rats. These effects may be attributed at least in part to the reduction of food intake as observed in the treated group, and are in line with our initial hypothesis that postulated a potential satiety-inducing effect of the BCA mixture. However, we cannot totally rule out the possibility that increase energy expenditure could have been involved in the limitation of weight gain and change of morphological parameters following BCA supplementation. The ability of berberine, citrus, and apple extracts to potentially increase thermogenic mechanisms, such as mitochondrial biogenesis and UCP-1 expression, is well-documented in the literature [21,22,48,49], For example, citrus polymethoxyflavones have been shown to enhance thermogenesis in brown adipose tissue (BAT) and inguinal white adipose tissue (iWAT) by activating AMPK/PGC1α signaling [17]. Similarly, apple polyphenols have been demonstrated to induce thermogenic adaptations in iWAT, possibly through the activation of the peripheral catecholamine synthesis–FGF21–PGC-1α cascade [18]. Moreover, berberine has been found to increase energy expenditure and limit weight gain in obese mice by promoting the expression of UCP1 and enhancing mitochondrial biogenesis [44]. While it’s plausible that the observed reduction in weight gain in the treated group could be attributed, at least in part, to increased energy expenditure mediated by these mechanisms, it’s essential to acknowledge that our study did not directly measure energy expenditure. Furthermore, previous studies have highlighted the potential of berberine, citrus, and apple extracts to mitigate weight gain through various metabolic pathways, including modulation of adipose tissue mass and lipid metabolism [17,18,44,45]. To sum up, while increased energy expenditure could be a plausible mechanism underlying the observed benefits on weight gain, our study design and existing literature support the view that the BCA supplementation exert beneficial effects through multiple metabolic pathways. Ideally energy expenditure should have been measured in order to accurately assess whether BCA may increase energy expenditure in addition to reduced food intake.

We next investigated the effects of BCA on whole-body metabolic homeostasis. As expected, Zucker rats exhibited increased fasting insulin levels as well as HOMA-IR when compared to the BCA-supplemented Zucker rats. Furthermore, untreated Zucker rats showed impaired glucose homeostasis as assessed by *per os* glucose tolerance tests. BCA attenuated this impairment only after 13 weeks supplementation indicating likely that this beneficial effect may be secondary to the reduction of body weight gain as the later was observed from the 8^th^ week supplementation. In contrast, although the relationship still needs to be clarified, our study failed to find any evidence that BCA-induced reduction of weight gain is linked with change of fasting total cholesterol and fasting triglycerides. The mild inhibition in weight gain observed in our study may be one possibility that could explain why nutraceutical supplementation did not limit the lipid disturbances occurring in treated-Zucker rats. Therefore, these findings suggested a specific positive effect of BCA on glucose tolerance and insulin resistance that could have been due to the large polyphenols content of BCA. However, they are at variance with the statement that apple and citrus polyphenols and berberine possess antilipidemic properties [36,49,50]. Whether dose of BCA up to 200 mg/kg and/or a long-term supplementation (>13 weeks) may significantly improve the lipid profile is unknown. Moreover, it is not unreasonable to postulate that antagonistic effects between the BCA ingredients could have been limited its delipidating ability at the used doses.

The functional alterations at the gut level associated with obesity, such as inflammation, permeability impairment and change of smooth muscle contractility have been poorly explored in both diet-induced obesity and specifically in obese Zucker rats. Our *in vitro* experiments revealed that colonic contractile effect following cholinergic stimulation (at the higher concentrations) was, although not significant, decreased in treated-Zucker rats. Some compounds present in BCA, notably polyphenols have been reported as signaling molecules that depress intestinal contractility [51]. Thus, although it is not necessarily valid to correlate the decreased colonic contraction of the BCA–supplemented rats with modulation of whole-animal gut transit, the potential decrease of colonic peristalsis that may occur under *in vivo* conditions supports the hypothesis that BCA supplementation may modulate, through cholinergic mechanism, the progress of the food bolus and likely the absorption of nutrients. However, it could be also suggested that the BCA-induced inhibition of colonic contractility may involve a role of gut microbiota *via* SCFA production. We evaluated the effect of butyrate and propionate on contractility of colonic segments obtained from untreated and BCA-treated Zucker rats. We found that carbachol-mediated contraction was increased after preincubation with propionate or butyrate. Studies investigating the effects of SCFA on intestinal contractility have shown different results depending on the muscle type studied [52,53]. The mechanism by which propionate or butyrate produces a relative potentiation of colonic response to carbachol is not readily apparent, but could involve specific local effects that may modify muscarinic receptor mechanisms or ion channels that involve calcium handling within the colonic smooth muscle cells linked to electrical coupling and contraction.

One of the objectives of the current research was also to investigate the potential benefit of BCA on the intestinal damage by exploring the change in colonic permeability using Ussing chambers. Under our experimental conditions, BCA supplementation significantly decreased the permeability of the distal colon supporting the hypothesis of a possible anti-inflammatory action of BCA that would likely result from an improvement of intestinal barrier function. In agreement with these results, berberine as apple or citrus ingredients and more broadly polyphenols have been shown to increase the expression of tight junction proteins in epithelial intestinal cells [54–56].

Metabolic syndrome and obesity are prominent risk factors contributing to the development of cardiovascular disease and are involved in the onset of endothelial dysfunction [57,58]. Obese Zucker rat is also characterized by the presence of endothelial dysfunction in addition to metabolic alterations (*i*.*e*. insulin resistance, dyslipidemia) [59–62]. However, our findings showed a great relaxant response to Ach in endothelium-intact aortic rings from control group (*with a near 100% relaxation rate*) which was comparable to that observed in the BCA-supplemented group. This is in line with our experience where the maximal relaxation in response to Ach in aorta from lean (Wistar) rats under the same conditions is typically in the range of 80–100%, excluding therefore the presence of endothelial dysfunction in response to muscarinic stimulation in 21-week-old obese Zucker rats. This finding aligns with previous literature reporting superior relaxation in obese Zucker rats compared to lean Zucker rats at 16-week-old [63] and in 32-week-old obese rats [60]. One of the hypotheses proposed to explain this unexpected result could be the intervention of adaptative mechanisms involving an increase in inducible nitric oxide synthase (iNOS) expression and/or in the cyclooxygenase (COX) pathway [63]. These putative mechanisms could have likely masked the beneficial effect of BCA mixture on endothelial function and could account for the decrease in maximal contraction to Phe observed in the BCA-ObZ group.

Conversely to the effect on the Ach-mediated relaxation, supplementation with BCA significantly enhanced insulin-mediated vasorelaxation, known to involve endothelial NO through the PI3K-Akt pathway, emphasizing specific action of BCA on this signaling. We also found that incubation of endothelium-intact aortic rings with eNOS cofactors (BH4 and L-arginine) improved relaxation only in untreated obese rats. The reason to account for this difference is not readily apparent. The fact that only aortic rings from control group were sensitive to cofactors supplementation supports the hypothesis that insulin signaling is impaired in obese Zucker rat. This impairment could have been attenuated under BCA supplementation and might explain why co-factor pretreatment failed to add any improvement in insulin relaxation in BCA-ObZ group.

Available research evidences have documented that the beneficial effects of citrus flavonoids, apple polyphenols and/or berberine on obesity and related disturbances could be mediated by mechanisms involving gut microbiota [34,64,65]. However, their combinatory effect on gut flora in obese Zucker rats is still unknown. We pursued this line of investigation to test the hypothesis that the potential anti-obesity effect of BCA might partially involve a positive modulatory effect of the gut microbiota in obese Zucker rats. We found that The BCA mixture reduced the proportion of Firmicutes and Actinobacteria, two phylum that promote energy absorption, letting us hypothesize that inhibition of energy absorption could be considered as another mechanism accounting for the reduction of weight gain observed in the treated Zucker rats. In addition, the significant increase of some genus such as *Akkermansia*, *Blautia*, *Lactobacillus* and *Muribaculum*, known to be important producers of SCFA [66] supports the theory that SCFA may be the link between gut microbiota and beneficial effect of BCA supplementation. SCFA have been shown to play an important role in the regulation of glucose and lipid metabolism [65,67,68] by mechanisms involving at least maintenance of the integrity of intestinal barrier and reduction of inflammation. However, in order to confirm that BCA mixture-induced reduction of colonic permeability can contribute to some of the metabolic improvement (i.e. decrease of insulin resistance) further studies are required.

There are some limitations to the present study. Firstly, the absence of the lean Zucker rat in the present study might be viewed as a limitation. However, we have considered that little information would be gained by including it because the experimental design has been conceived to evaluate the effects of BCA directly in the obese Zucker rats, and additional experiments with their lean counterparts are not essential to the message of the present paper. Secondly, we studied a nutraceutical mixture of berberine and plant extracts containing several ingredients at different concentrations acting alone or in synergy and/or antagonism. Further works would be required to assess in our experimental conditions the individual contribution of each component of the mixture to the global activity. Thirdly, whether a prolonged supplementation (>13 weeks) may induce a more powerful and lasting effect on obesity and related disorders in obese Zucker rat, therefore, the results must be interpreted with caution.

Our data provide the first evidence to our knowledge for a beneficial effect of nutraceutical mixture with berberine and extracts of apple and citrus for preventing weight gain, improving glycemic control and insulin sensitivity in obese Zucker rat. Both aortic and colonic findings support the hypothesis that long-term BCA supplementation has vascular and gut protective potential that warrants further investigation. As a significant increase of SCFA-producing species (*Akkermansia municiphila*, *Blautia*, *Lactobacillus* and *Muribaculum*) was observed after BCA supplementation, it is tempting to suggest that the metabolic and functional improvements observed could be explained, at least in part by a positive modulatory effect of BCA on gut microbiota. Finally, we believe that long-term BCA supplementation has the potential of a promising therapeutic option in metabolic and vascular pathology linked to the obesity, nevertheless further research is needed for better understanding the health-promoting effects of BCA used as a dietary supplement.

## Supporting information

S1 Fig Effect of BCA mixture on liver in obese Zucker rats, quantification of area of steatosis (A) and area of fibrosis (B). (DOCX)

S2 Fig Effect of BCA mixture on cecum size in obese Zucker rats, representative photograph (A) and Cecum weight as a percentage of body weight (B). (DOCX)

S3 Fig Evolution in gut microbiota composition between week 0 (A&B) and week 13 (C&D). (DOCX)

## Abbreviations

Ach: Acetylcholine
ALAT: Alanine aminotransferase
ASAT: Aspartate aminotransferase
AUC: Area under the curve
AVS: Amplicon variant sequences
BCA: Berberine, Citrus and Apple
BCA-ObZ: BCA-Treated Obese Zucker
BMI: Body Mass Index
CCRCs: Cumulative concentration-response curves
HDL: High-density lipoprotein cholesterol
HOMA: Homeostasis model assessment
Ins: Insulin
IR: Insulin resistance
L-Arg: L-arginine
LDL: Low-density lipoprotein cholesterol
LME: Linear mixed-effects
NLME: Nonlinear mixed-effects
NO: Nitric oxide
OGTT: Oral Glucose Tolerance Test
Phe: Phenylephrine
SCFA: Short-chain fatty acids
SEM: Standard error of the mean
U-ObZ: Untreated Obese Zucker
