# Supplementary material for: Preventive effects of a nutraceutical mixture of berberine, citrus and apple extracts on metabolic disturbances in Zucker fatty rats
Source: PLoS One. 2024 Jul 26;19(7):e0306783. doi: 10.1371/journal.pone.0306783 (PMC11280259; doi:10.1371/journal.pone.0306783)
Supplement: S1 Fig — (DOCX) [file pone.0306783.s001.docx]

*
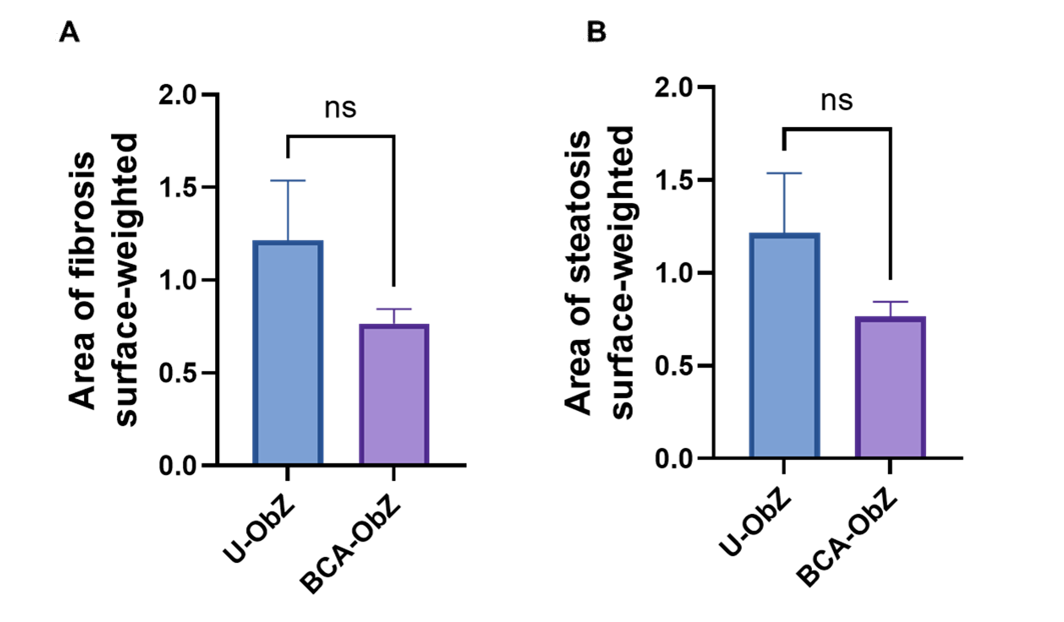
*

Supplementary Figure 1: Effect of BCA mixture on liver in obese Zucker rats, quantification of area of steatosis (A) and area of fibrosis (B). Data are expressed as mean ± SEM. The Mann-Whitney test was used for statistical analysis. n=8 for each group. U-ObZ: Untreated Obese Zucker; BCA-ObZ: BCA-Treated Obese Zucker; ns: not significant.
