# Supplementary material for: Preventive effects of a nutraceutical mixture of berberine, citrus and apple extracts on metabolic disturbances in Zucker fatty rats
Source: PLoS One. 2024 Jul 26;19(7):e0306783. doi: 10.1371/journal.pone.0306783 (PMC11280259; doi:10.1371/journal.pone.0306783)
Supplement: S2 Fig — (DOCX) [file pone.0306783.s002.docx]

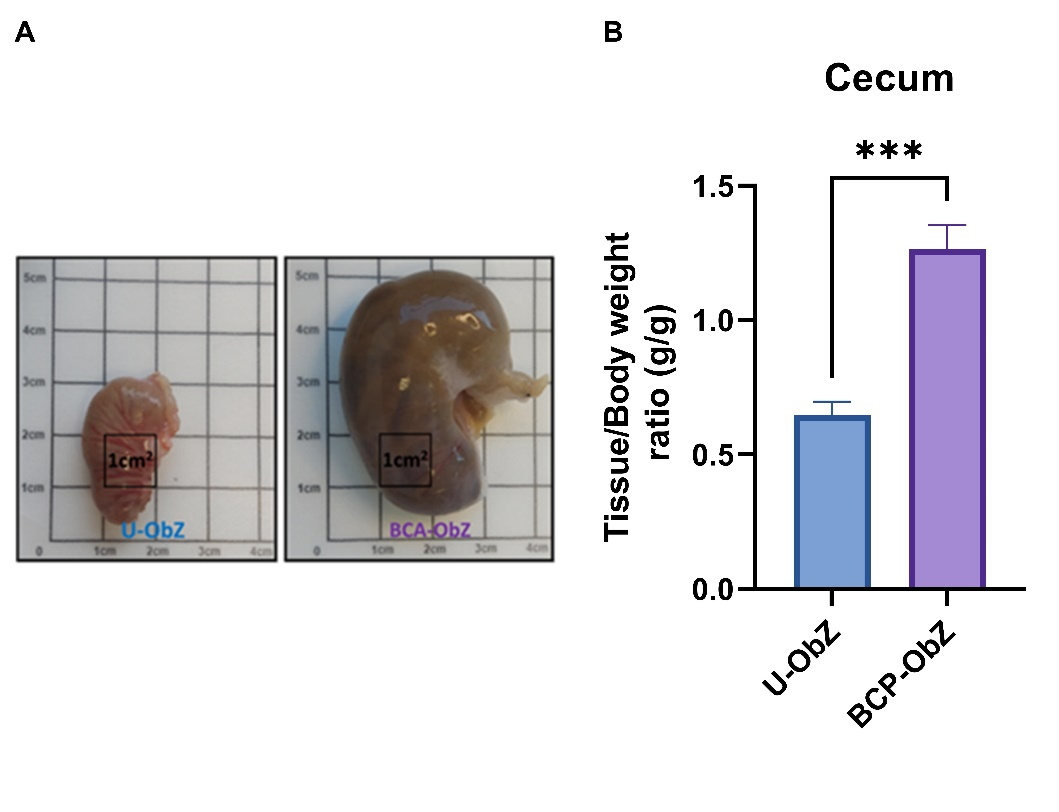


Supplementary Figure 2: Effect of BCA mixture on cecum size in obese Zucker rats, representative photograph (A) and Cecum weight as a percentage of body weight (B). Data are expressed as mean ± SEM. The Mann-Whitney test was used for statistical analysis. n=8 for each group. *** p<0.001 BCA-ObZ vs. U-ObZ. U-ObZ: Untreated Obese Zucker; BCA-ObZ: BCA-Treated Obese Zucker.
