# Supplementary material for: Preventive effects of a nutraceutical mixture of berberine, citrus and apple extracts on metabolic disturbances in Zucker fatty rats
Source: PLoS One. 2024 Jul 26;19(7):e0306783. doi: 10.1371/journal.pone.0306783 (PMC11280259; doi:10.1371/journal.pone.0306783)
Supplement: S3 Fig — (DOCX) [file pone.0306783.s003.docx]

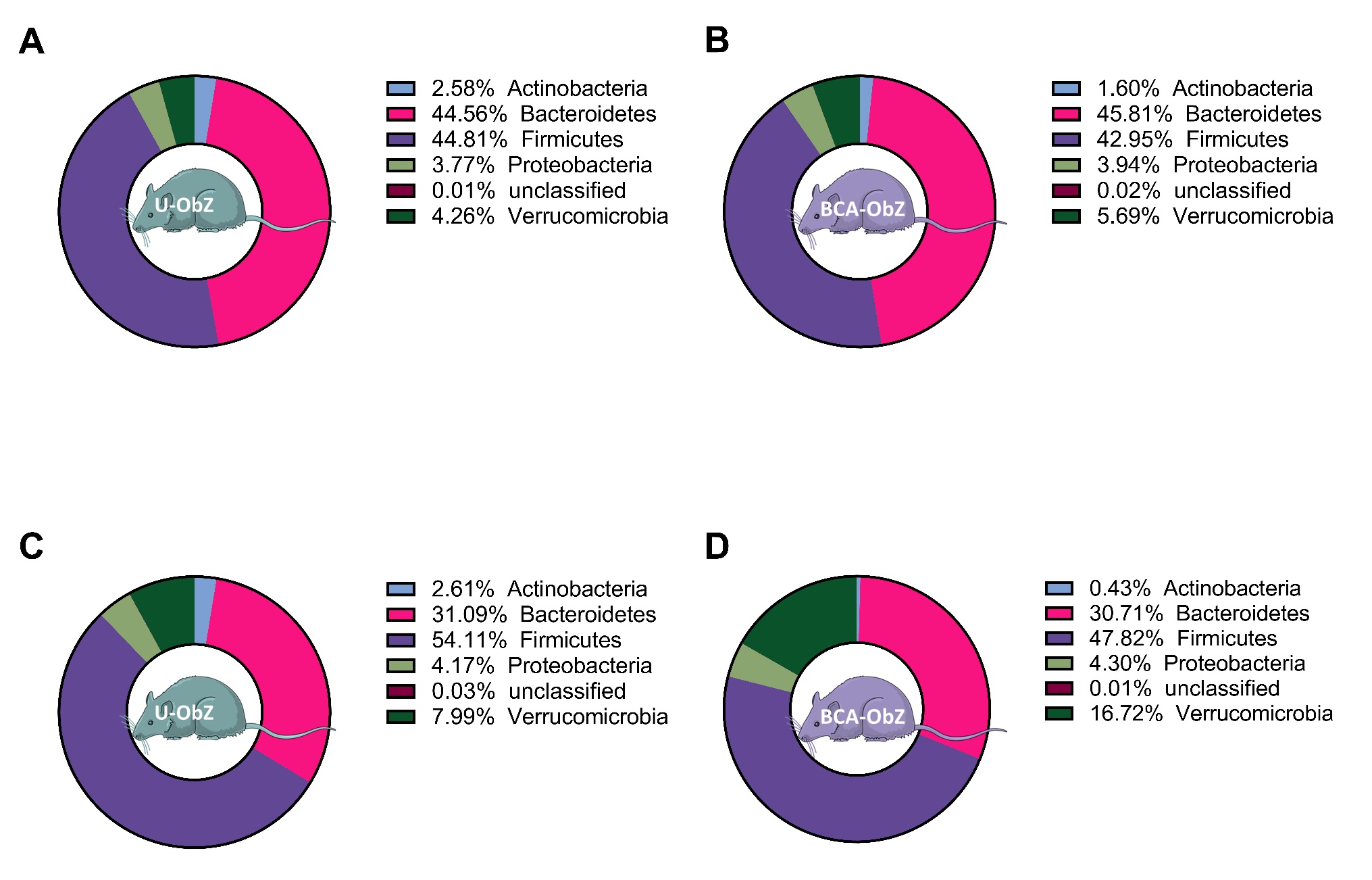


Supplementary Figure 3: Evolution in gut microbiota composition between week 0 (A&B) and week 13 (C&D). n=8 for each group. U-ObZ. U-ObZ: Untreated Obese Zucker; BCA-ObZ: BCA-Treated Obese Zucker.
